# Patterns in conflict dynamics in Yemen and Syria

**DOI:** 10.1098/rsos.260467

**Published:** 2026-11-04

**Authors:** Samar Moussa Abdou, Neil F. Johnson

**Affiliations:** ^1^ Department of Statistics, The George Washington University, Washington, DC, USA; ^2^ Department of Physics, The George Washington University, Washington, DC, USA

**Keywords:** conflict patterns, extreme events, dynamics

## Abstract

Conflict fatalities tend to follow heavy-tailed statistical distributions. A 2005 fusion–fission theory predicts that, when armed groups operate through dynamically forming and fragmenting clusters, the number of fatalities per event should be approximated by a power-law distribution with exponent α near 2.5, with deviations reflecting the relative robustness of larger versus smaller fighter clusters. We use Armed Conflict Location and Event Data (ACLED) event-level fatality data to estimate α in recent Yemen and Syria conflict periods during 2023–2025. These conflict event datasets are small, incomplete and imperfect and only produce very crude approximations to a power law; nonetheless, the best-fit α values occupy the range 2.5<α<3.5 across most periods, which is broadly consistent with predominantly fragmented or weakly consolidated armed-group organization. Temporary reductions in α are broadly consistent with episodes in which larger coordinated clusters become more consequential. More rigorous testing will require improved datasets to become available in the future.

## 1. Introduction

The study of human conflicts and terrorism (no matter how defined) has a long and distinguished history across a wide variety of disciplinary perspectives [1–11]. Irrespective of their myriad causes, conflicts tend to exhibit highly irregular fatality patterns [12–33]. Instead of following thin-tailed statistical models such as a typical bell-curve Gaussian, conflict fatalities routinely form heavy-tailed distributions in which rare but extremely deadly events occur far more often than everyday assumptions about the randomness of war would predict [12,16,21,26,30,31]. This empirical regularity suggests that severe casualty events may arise from collective organization and interaction mechanisms rather than from independent random fluctuations alone.

A recent study [21] provides a physics-based theory that can quantitatively and mechanistically explain such fatality patterns in the Israel–Palestine regional conflicts, including the most recent war in Gaza that started in October 2023. It employs the original mathematical fusion–fission model for conflict and terrorism first published in 2005 on the public arXiv server [12,22,30,32]. The theory’s concrete prediction of α being in the vicinity of 2.5 for any given conflict has since been verified in multiple out-of-sample empirical studies on (i) conflicts that started after the prediction appeared publicly in 2005, and also on (ii) conflict datasets that were produced after this same date. Each class of tests was, hence, strictly out of sample [12,21,22,24–27,30–33]. The theory was also recently shown to explain the distribution of total-casualties-per-war across wars [12,26,28,29,31], as well as casualties by region within a given country’s conflict [33], and also patterns across online spaces that can nurture extremist support [27].

The theory’s fusion–fission model is deliberately minimal. It purposely averages over the many sociological, political, technical, geographical and economic details that individually play a narrative role but whose combined contribution crudely cancels in terms of determining the fatality distribution. The theory’s purpose is not to replace such conflict narratives, nor to replace the detailed information available to intelligence agencies, field researchers, journalists or humanitarian monitors. Rather, its purpose is to provide a reproducible and theory-grounded aggregate diagnostic that can be applied to open event data. By focusing on the kinetics of how humans fight collectively, the model yields fusion–fission dynamical equations that can be solved using standard algebra, producing concrete and robust mathematical predictions for the distribution of fatalities per event and its expected power-law exponent α [22].

The independent empirical study discussed in [21] confirms that the fusion–fission fighter mechanism that forms the core of the theory is realistic at the level of armed-group organization: fighter groups undergo a continual process in which they fuse into larger coordinated clusters and fragment into smaller clusters under pressure [22,32]. It can then be shown mathematically [22] that when fusion and fission balance in the model’s stationary regime, the distribution of fighter cluster sizes follows a power law with exponent α=2.5+δ, where −1≲δ≲1 depends on the relative robustness of larger versus smaller fighter clusters. The amount by which an empirical α value deviates from 2.5 therefore offers a coarse statistical indication of the relative robustness of larger clusters in that conflict [12,22].

Specifically, the model predicts that a conflict in which larger clusters are relatively more robust—more akin to a formal army formation on a traditional battlefield—should have an empirical α closer to 1.5 (i.e. δ→−1). This is because larger robust clusters that can produce proportionally larger damage make high-fatality events more probable, producing a fatter tail. In the opposite limit, in which the conflict features many smaller fighter clusters and hence smaller numbers of fatalities per event—more akin to an extreme guerrilla war or insurgency—the empirical α should be closer to 3.5 (i.e. δ→1). This steeper tail means that events with large numbers of fatalities remain possible but are less probable. When the two limits are balanced, the prediction is that the empirical α should be approximately 2.5.

This paper contributes to the current conflict literature in two more limited and explicitly exploratory ways. First, we analyse event-size distributions for recent Yemen and Syria data. Neither country has been the focus of previous event-level applications of this specific fusion–fission exponent analysis to our knowledge, while both remain important contemporary conflict settings. Second, we ask whether the temporal evolution of the best-fit empirical α values might help provide an interpretable open-data summary of changes in the distribution of event severity. We do not claim that α reveals detailed order-of-battle information that would already be available from classified or field intelligence. We also recognize that on small, incomplete and imperfect datasets such as Yemen and Syria, the distribution is at best a very noisy version of an approximate power law—yet the best-fit α can still be calculated in the same rigorous quantitative way. Hence, the overall value of the approach is that it offers a low-dimensional, reproducible and theoretically interpretable summary of event-level fatality distributions, allowing comparisons across conflicts and across periods within a conflict. Despite the empirical caveats owing to the limitations of the datasets, a downward drift in α can, in principle, be interpreted mechanistically as a movement towards a heavier-tailed fatality distribution, which is consistent with a greater role for larger or more robust operational clusters. Our present two-case study cannot establish that such movements predict the exact timing of tipping points, such as the collapse of a regime’s organized military capacity. Establishing that stronger claim would require pre-registered tests across a broader set of conflicts, clearly defined outcome events and rolling-window procedures evaluated before those outcomes occur. We therefore treat the early warning interpretation here as a hypothesis-generating result rather than as a validated forecasting tool.

The power-law form is used here because it is the explicit prediction of the fusion–fission theory, not because a power law must always be the single best-fitting member of a large menu of candidate heavy-tailed distributions. The power-law distribution has a theoretical grounding for conflicts; it is simpler than many other candidate distributions, and the estimated α values have intuitive interpretations. We recognize that in empirical data, a more flexible distribution can sometimes outperform a simpler theory-driven model. That fact does not by itself invalidate α-tracking as a useful diagnostic, provided the fitted power-law tails are not visually or statistically completely implausible and provided the interpretation is framed as an approximate, model-based summary. We report goodness-of-fit diagnostics, include visual complementary cumulative distribution function (CCDF) checks and interpret the estimates very cautiously.

Both the Yemen and Syria conflicts include periods of dispersed, localized violence and periods of sharper military or political shock. We ask whether such shifts might appear as temporary changes in the best-fit exponent α. In both conflicts, the best-fit exponent α begins near 3.0 in the periods we analyse and later moves closer to 2.5 during periods associated with more severe fatality events. Our results are summarized in figures 1 and 2 and tables 1 and 2. The interpretation we draw is cautious: these patterns are consistent with a common statistical life cycle in which a fragmented fatality regime is followed by a period with a heavier tail, and then by a possible post-crisis readjustment. They do not by themselves prove the underlying organizational mechanism or establish predictive timing.

**Figure 1.**
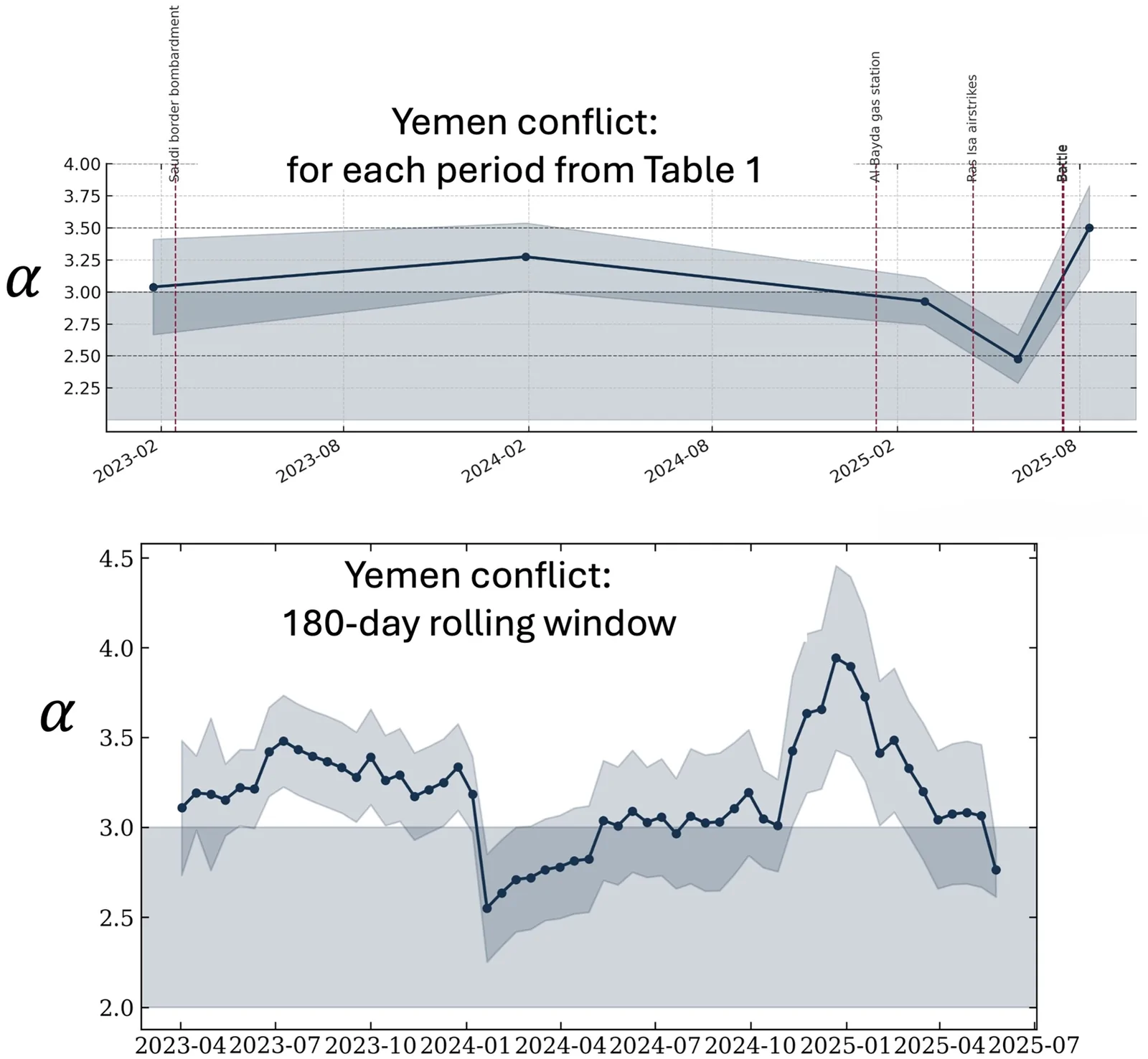
Power-law exponent α estimates for conflict fatalities during the Yemen conflict. The upper panel shows period-based estimates corresponding to the events in table 1; the lower panel shows rolling estimates using a 180-day window. Solid lines denote maximum-likelihood estimates with 95% confidence intervals. As a guide, the shaded band marks α∈[2.0,3.0]. Downward movements in α indicate heavier fitted tails and hence a greater relative probability of high-fatality events under the model, but the rolling curve is exploratory and should not be interpreted as a validated forecast of specific transitions.

**Figure 2.**
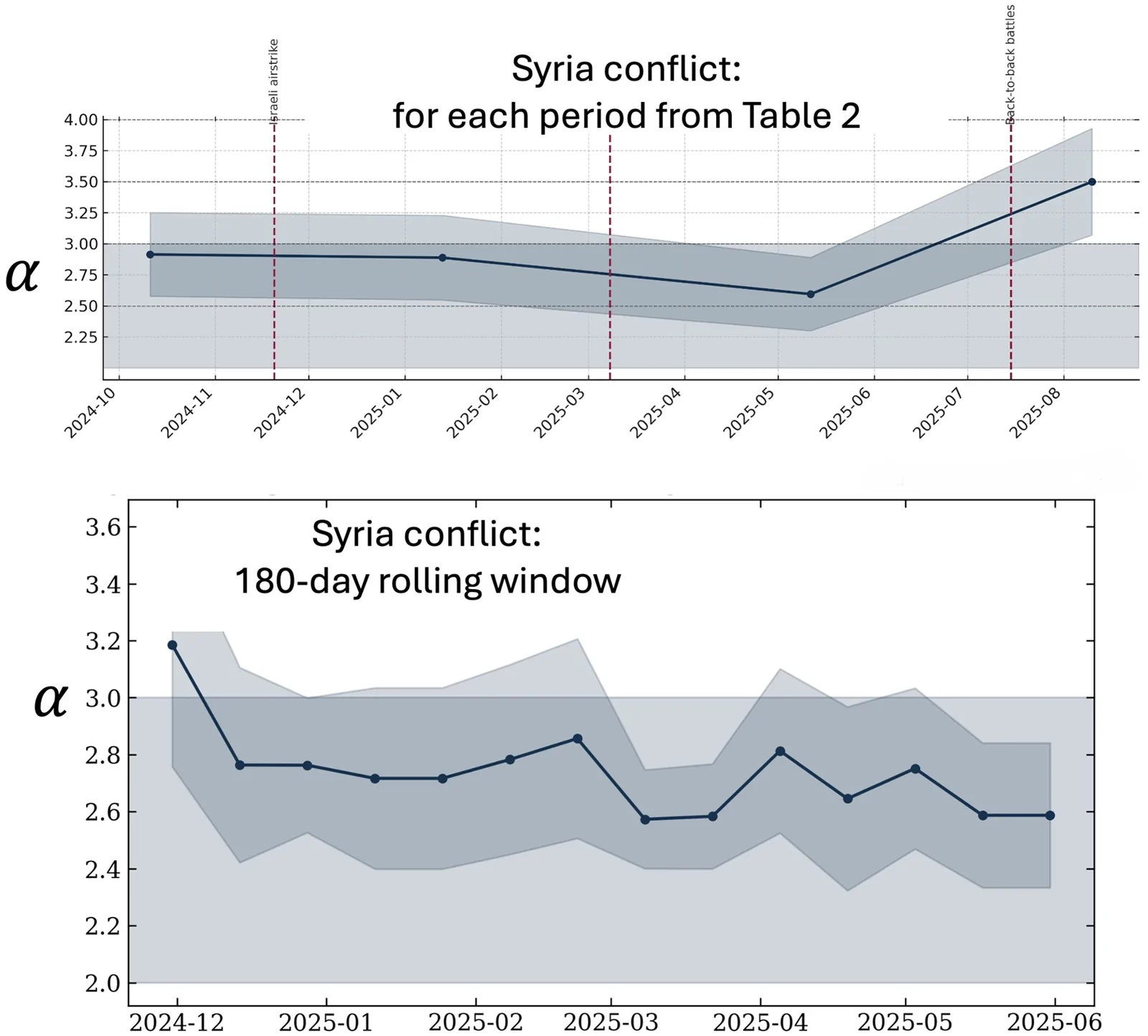
Power-law exponent α estimates for conflict fatalities during the Syria conflict. The upper panel shows period-based estimates corresponding to the events in table 2; the lower panel shows rolling estimates using a 180-day window. Solid lines denote maximum-likelihood estimates with 95% confidence intervals. As a guide, the shaded band marks α∈[2.0,3.0]. The period-level and rolling-window estimates need not coincide exactly because they use different averaging windows in a non-stationary conflict system.

**Table 1.** Power-law exponent estimates for different periods attached to specific events within the Yemen conflict (2023–2025). The reported $p_{\mathrm{KS}}^{\mathrm{asym}}$ values are approximate descriptive one-sample KS p-values computed from the reported KS distance and tail size; because $x_{\min}$ and α are estimated, they should be interpreted conservatively and not as exact bootstrap goodness-of-fit probabilities. The uncertainty associated with these estimates is substantial because of limited tail observations and known limitations of conflict-event reporting. However, this is a natural consequence of the relatively few events and the quality of the event data reporting, which is separate from our study and is beyond our control.

period	event	α	$x_{\min}$	tail n	KS	$p_{\mathrm{KS}}^{\mathrm{asym}}$	95% CI
2023-01-02 to 2023-02-15	Saudi border bombardment (approx. 130)	3.037	2.0	115	0.313	$1.6\times 10^{-10}$	(2.66, 3.41)
2023-02-15 to 2025-01-11	Al-Bayda gas station (approx. 40)	3.273	6.0	286	0.178	$2.1\times 10^{-8}$	(3.00, 3.53)
2025-01-11 to 2025-04-17	Ras Isa air strikes (approx. 80)	2.925	2.0	421	0.308	$5.9\times 10^{-36}$	(2.74, 3.10)
2025-04-17 to 2025-07-15	Battle (approx. 100)	2.475	2.0	236	0.296	$8.0\times 10^{-19}$	(2.28, 2.66)
2025-07-16 to end	Post-2025-07-16	3.500	1.0	223	0.650	$1.4\times 10^{-92}$	(3.17, 3.82)

**Table 2.** Power-law exponent estimates for different periods attached to specific events within the Syria conflict (2024–2025). The $p_{\mathrm{KS}}^{\mathrm{asym}}$ values are approximate descriptive one-sample KS p-values and should be interpreted conservatively for the same reasons as given in table 1. The statistical support for our results is again lower than desirable. However, this is a natural consequence of the relatively few events and the quality of the event data reporting, which is separate from our study and is beyond our control.

period	event	α	$x_{\min}$	tail n	KS	$p_{\mathrm{KS}}^{\mathrm{asym}}$	95% CI
2024-09-01 to 2024-11-20	Israeli air strike (approx. 105)	2.913	4.0	124	0.209	$3.1\times 10^{-5}$	(2.57, 3.25)
2024-11-20 to 2025-03-08	Massacres (approx. 80–94)	2.887	11.0	118	0.092	0.255	(2.54, 3.22)
2025-03-08 to 2025-07-15	Back-to-back battles (approx. 100)	2.594	13.0	112	0.165	0.0039	(2.29, 2.88)
2025-07-15 to end	Post-2025-07-15	3.498	5.0	130	0.277	$2.6\times 10^{-9}$	(3.06, 3.92)

## 2. Methodology

This study analyses fatality data from Armed Conflict Location and Event Data (ACLED) for Yemen (January 2023–September 2025) and Syria (January 2023–August 2025). Only events with at least one recorded fatality were included, because the statistical behaviour of interest concerns the distribution of event sizes conditional on a fatal event, not the occurrence frequency of non-fatal events. To capture possible structural changes, each country’s data were segmented into periods reflecting major military or political transitions identified from the conflict chronology and ACLED event records [1,34]. For Yemen, these segments correspond to the Saudi border bombardment, the Al-Bayda explosion, the Ras Isa terminal strikes and the July 2025 battles. For Syria, the segmentation reflects a prolonged stability period, escalation beginning in late 2024, a collapse-transition period through mid-2025 and a post-collapse environment. For comparison, we also analyse a 180-day rolling window that is independent of these period labels.

### 2.1. Choice of event dataset

We use ACLED because it provides event-level, near-real-time conflict data across political violence and protest categories and is therefore suitable for a 2023–2025 analysis [1,34]. We recognize that the Uppsala Conflict Data Program Georeferenced Event Dataset (UCDP GED) provides an important alternative event dataset with a different coding philosophy and is widely used for organized-violence research [9,35]. We do not mix ACLED and UCDP observations in the same fits because differences in event definitions, source rules, geocoding and fatality coding can change the tail of the event-size distribution. A full robustness test would therefore require repeating the same workflow on UCDP GED once the relevant time windows and comparable event definitions are fixed. We regard that as a valuable future test rather than a simple substitution.

For Yemen, the Yemen Data Project provides detailed air-raid data that are especially relevant for studying mechanisms in which large groupings become vulnerable to aerial attack. However, its main public Saudi-led coalition air-raid dataset covers March 2015–April 2022, before the 2023–2025 window analysed here [36]. It is therefore not a direct substitute for the ACLED data used in the present fits. It is instead a potentially valuable supplementary source for future mechanism-oriented studies, particularly if comparable air-strike data are extended to the periods analysed here.

### 2.2. Fusion–fission model and exponent interpretation

The theoretical motivation for this analysis comes from the fusion–fission model of armed-group organization [12,21,22]. In this model, clusters of fighters continually fuse and fragment as they build up and then scatter when sensing danger. Describing this dynamical fusion–fission process mathematically generates the prediction that α will have a value of 2.5+δ, where −1≲δ≲1 and the precise value depends on the relative robustness of larger versus smaller fighter clusters in a given conflict. This result can be derived from the following equations for the rate of change of the number of fighter clusters $n_{s}$ that contain s fighters (i.e. size s=1,2,…,N):

(2.1) $$\begin{array}{rl} {\dot{n}}_{s}= & \left[\begin{array}{c} \text{number of fighter clusters of}\\ \text{size}\;s\;\text{being formed}\\ \text{at time}\;t\;\text{through}\\ \text{fusion (or fission)} \end{array}\right]\;-\;\left[\begin{array}{c} \text{number of fighter clusters of}\\ \text{size}\;s\;\text{being removed}\\ \text{at time}\;t\;\text{through}\\ \text{fission (or fusion)} \end{array}\right]\\ = & \;\left[\frac{F}{2}\sum_{\begin{array}{c} 0⩽i⩽s\\ 0⩽j⩽s \end{array}}^{i+j=s}(ij{)}^{1+\delta }\frac{n_{i}n_{j}}{N^{2}}+\delta_{1s}\frac{\varphi_{f}}{2}\sum_{j=1}^{\infty }j^{2+\delta }\frac{n_{j}}{N}\right]-\left[\left(F\sum_{i=1}^{\infty }i^{1+\delta }\frac{n_{i}}{N}+\frac{\varphi_{f}}{2}\right)s^{1+\delta }\frac{n_{s}}{N}\right]. \end{array}$$

Here, $\varphi_{f}$ describes the fighter-cluster fission probability, while F describes the fighter-cluster fusion probability; N is the number of fighters involved in this fusion–fission process, and, for simplicity, we have suppressed additional labels denoting organizations, ideologies or factions.

We do not repeat the already-published derivation of α=2.5+δ from equation (2.1), but instead refer interested readers to [12,21], where the calculation is given in detail. We provide the following interpretation in order to convey its meaning. The first bracket in equation (2.1) includes all the ways that the number of fighter clusters of size s can increase at a given time t: through fusion of smaller fighter clusters, or, for s=1, through the fission of a larger cluster into small fragments. The second bracket includes all the ways that the number of fighter clusters of size s can decrease: through fission of a cluster of size s, or through fusion into a larger cluster. A cluster of size s=i+j is created by fusion of two smaller clusters i and j; a cluster of size s is lost either by fission or by fusion with another cluster.

The result α=2.5+δ is robust to several variations in the model set-up (see appendix A). Its mathematical validity requires that post-fission fragments are small relative to the pre-fission cluster size; complete fragmentation into single fighters is not required. Temporal variation of N only needs to be slow compared with the fusion–fission dynamics. If the fighter force comprises one type of fighter, F is a number that depends on the average heterogeneity of those fighters [21]; with D>1 adversarial types, F becomes a matrix; see [21] for the full multi-type equations.

Akin to reaction kinetics, we take the number of fighters s in a cluster as determining, on average, the total number of casualties x in an event involving that cluster. Here, x can include fighters on either side and civilians, and it may be a constant multiple or fraction of s. If the rate at which fighter clusters are involved in events is approximately independent of cluster size, the distribution $n_{s}$ has the same mathematical form as the casualty distribution $n_{x}$, i.e. the number of events with x casualties. Hence, the power law $n_{s}∼s^{-\alpha }$ for fighter-cluster sizes also describes the distribution of casualties per event on average. This approximation is deliberately coarse. Real event severity is affected by weapon type, target type, civilian exposure, reporting bias and event aggregation.

Taken together, the 2005 fusion–fission model motivates use of the approximate power-law distribution

(2.2) $$p(x)=\frac{\alpha -1}{x_{\min}}{\left(\frac{x}{x_{\min}}\right)}^{-\alpha },\;x\geq x_{\min}.$$

A higher α does not mean that high-fatality events are impossible; it means that they are less probable relative to low-fatality events. Conversely, lower α corresponds to a heavier tail in which high-fatality events are more common under the fitted model.

### 2.3. Power-law fitting and diagnostics

We choose the framework of power-law distributions because they have theoretical grounding for conflicts, they are simpler than many other candidate distributions and the estimated α values have intuitive interpretations. We obtain a best-fit estimate of α using the Clauset–Shalizi–Newman maximum-likelihood method [16]. The lower cut-off $x_{\min}$ is selected to minimize the Kolmogorov–Smirnov (KS) distance between the empirical data and the fitted model. Given n tail observations $x_{i}\geq x_{\min}$, the log likelihood for the continuous power law is

(2.3) $$\ln L(\alpha )=n\ln(\alpha -1)-n\ln x_{\min}-\alpha \sum_{i=1}^{n}\ln\left(\frac{x_{i}}{x_{\min}}\right),$$

and maximizing this expression yields the estimator

(2.4) $$\hat{\alpha }=1+n{\left[\sum_{i=1}^{n}\ln\left(\frac{x_{i}}{x_{\min}}\right)\right]}^{-1}.$$

Goodness-of-fit is summarized using the KS statistic

(2.5) $$D=\max_{x}|S(x)-P(x)|,$$

where S(x) is the empirical cumulative distribution function and P(x) is the fitted model. We also report an approximate one-sample asymptotic KS p-value, denoted $p_{\mathrm{KS}}^{\mathrm{asym}}$, as a descriptive diagnostic. Because α and $x_{\min}$ are estimated from the same data, and because the data include rounding and event-coding noise, these values should not be interpreted as exact Clauset-style bootstrap p-values. The CCDF,

(2.6) $$P(X\geq x)={\left(\frac{x}{x_{\min}}\right)}^{-(\alpha -1)},$$

is further described in appendix B. These plots are intended to indicate visually whether the power-law tails are plausible at the period level, not to imply that every rolling-window fit is equally reliable.

### 2.4. Rolling windows

In addition to period-specific fits, we compute rolling estimates of α using overlapping 180-day windows (figures 1 and 2). These rolling estimates should be interpreted as local, smoothed summaries of a non-stationary process. They are not expected to coincide exactly with the period-level fits because the two procedures estimate different quantities: the period-based fits aggregate data over historically defined intervals, whereas the rolling-window fits use a fixed-length moving window that can straddle multiple phases.

The rolling-window series can suggest changes in tail heaviness, but the derivative dα/dt is noisy because adjacent windows overlap, tail sample sizes fluctuate, $x_{\min}$ can jump and a few large events can influence the estimate. A sustained decline in α is therefore best interpreted as a candidate signal that high-fatality events have become more probable under the fitted model. It is not, in the present paper, a validated method for predicting a specific transition date. A formal early warning test would require a wider conflict sample, pre-specified outcome events, out-of-sample evaluation and comparisons with baseline indicators.

## 3. Results: Yemen (2023–2025)

Figure 1 and table 1 suggest a coarse progression in Yemen’s event-size distribution between 2023 and 2025. During the initial periods, the period-level estimates are above 3.0, indicating a relatively steep fitted tail. Under the model, this is consistent with a setting in which high-fatality events are statistically less frequent, and violence is comparatively fragmented. This does not mean that large events cannot occur; it means that they are less common relative to smaller fatality events.

The February 2023 Saudi border bombardment is a major shock, but the period-level α values do not immediately move towards 2.5. This is consistent with the possibility that the shock did not, by itself, reorganize the broader event-size distribution into a heavier-tailed regime. The rolling estimates show more short-term variation, as expected from their overlapping windows and different averaging structure.

After the January 2025 Al-Bayda gas station explosion and the subsequent high-fatality events, the fitted exponent declines towards α≈2.9. During the Ras Isa air strike and April–July 2025 battle periods, the period-based estimate moves closer to 2.5. This is consistent with a heavier-tailed event-size distribution and with a larger relative role for events involving more robust or more consequential operational clusters. The strongest period-level shift occurs in April–July 2025, where α=2.475 with a 95% confidence interval of (2.28,2.66).

The final Yemen period has α=3.500, which would suggest a return to a steeper tail if the fit were reliable. However, this period also has the largest KS distance and the smallest approximate KS p-value in table 1. We therefore treat this rebound cautiously. It may reflect redispersion after a peak phase, but it may also reflect data-quality problems, event aggregation or the instability of fitting a power law to that period’s tail.

Overall, the Yemen results support a cautious interpretation: the fitted tail is relatively steep during much of the earlier window, becomes heavier around the 2025 escalation periods and may steepen again afterward. This is compatible with the fusion–fission interpretation but is not, by itself, a proof of the underlying mechanism.

## 4. Results: Syria (2023–2025)

Figure 2 and table 2 suggest that Syria’s event-size distribution also moves through a coarse sequence of tail regimes. The period-level estimates before March 2025 are near α≈2.9, indicating a moderately steep tail. The March–July 2025 period then has α=2.594, which is closer to the model’s balanced value of 2.5 and indicates a heavier fitted tail.

We interpret this pattern cautiously. It is consistent with a period in which high-fatality events become more probable under the fitted distribution. It is also consistent with the fusion–fission picture in which larger or more robust operational clusters become more consequential. However, it is not sufficient to infer the detailed organization of Syrian armed actors, nor does it provide a validated prediction of regime collapse or any other specific tipping point.

The post-July 2025 period has a higher estimate, α=3.498, suggesting a possible return to a steeper tail. This result should again be read in light of the diagnostics: the approximate KS p-value is small, and the rolling-window estimates do not show the same sharp rebound because the 180-day windows smooth across adjacent periods. Thus, the period-level fit and the rolling-window curve tell related but not identical stories. The period-level estimates are best used for coarse comparisons across historically defined phases, while the rolling-window estimates are best used as exploratory local summaries.

Overall, the Syria results are suggestive of a move towards a heavier-tailed fatality distribution in early-to-mid 2025 followed by possible readjustment. This is broadly comparable to the Yemen pattern, but the similarity is statistical and should not obscure the very different political and military contexts of the two conflicts.

## 5. Discussion

The fitted exponent α should be understood as a statistical tracer of event-size distributions, not as a direct observation of armed-group structure. Intelligence and field reports can identify specific units, command relationships, supply chains and battlefield movements in a way that an aggregate exponent cannot. Conversely, an exponent estimated from open event data can summarize the tail of the fatality distribution in a reproducible way, allowing comparisons across time windows and cases even when detailed organizational information is unavailable or contested. In this paper, ‘robustness’ is therefore used in an operational statistical sense: lower α implies a heavier fitted tail, which is consistent with a larger role for events that can produce many fatalities. The fusion–fission theory provides one mechanism that links such a tail to more robust larger clusters of fighters. Other mechanisms, including weapon changes, targeting choices, event aggregation and reporting practices, may also alter the tail. This is why the causal interpretation must remain cautious.

The discrepancy between period-level and rolling-window estimates is not a contradiction but a consequence of non-stationarity and different averaging schemes. Period-level fits answer the question: what is the best-fit exponent α for a historically defined interval? Rolling-window fits answer the question: how does a local best-fit α estimate change as the window moves? If the conflict changes rapidly, the two approaches can disagree. We therefore place more weight on period-level fits for coarse phase comparisons and treat rolling-window changes as exploratory indicators that require validation in larger samples.

## 6. Conclusion

We chose for our study the framework of power-law distributions because they have theoretical grounding for conflicts, they are simpler than many other candidate distributions and the estimated α values have intuitive interpretations. Doing this, we were able to provide an exploratory quantitative perspective on recent conflict dynamics in Yemen and Syria. Using a theory-driven power-law exponent derived from the fusion–fission model, we find that most fitted values lie between 2.5 and 3.5, with temporary movement towards lower α during periods associated with more severe fatality events. In the model, such lower values correspond to heavier fitted tails and are consistent with a greater relative role for larger or more robust operational clusters.

There is much room for future improvement in our study in terms of improving the quality of the data and deepening what our analysis tells us. Several limitations should be emphasized. The statistical support for our results is lower than desirable. However, this is a natural consequence of the relatively few events and the quality of the event data reporting, which is separate from our study and is beyond our control. In addition, we do not claim that α alone reveals the detailed organization of fighting forces or predicts the exact timing of conflict tipping points. The evidence here consists of two recent cases, and the period definitions are informed by known events. The early warning interpretation should therefore be treated as a hypothesis to be tested rather than as a demonstrated forecasting result. A rigorous future test would need to evaluate rolling α estimates prospectively across many conflicts, specify escalation outcomes in advance, compare against baseline indicators and test robustness across ACLED, UCDP GED and other relevant event datasets.

Despite these limitations, the Yemen and Syria results suggest that α can be a useful theory-grounded summary of how the severity distribution changes over time. Its main contribution is not to replace detailed conflict knowledge but to provide a compact and reproducible statistical lens through which organizational dynamics can be compared across cases and periods.

## Figures and Tables

**Figure 3.**
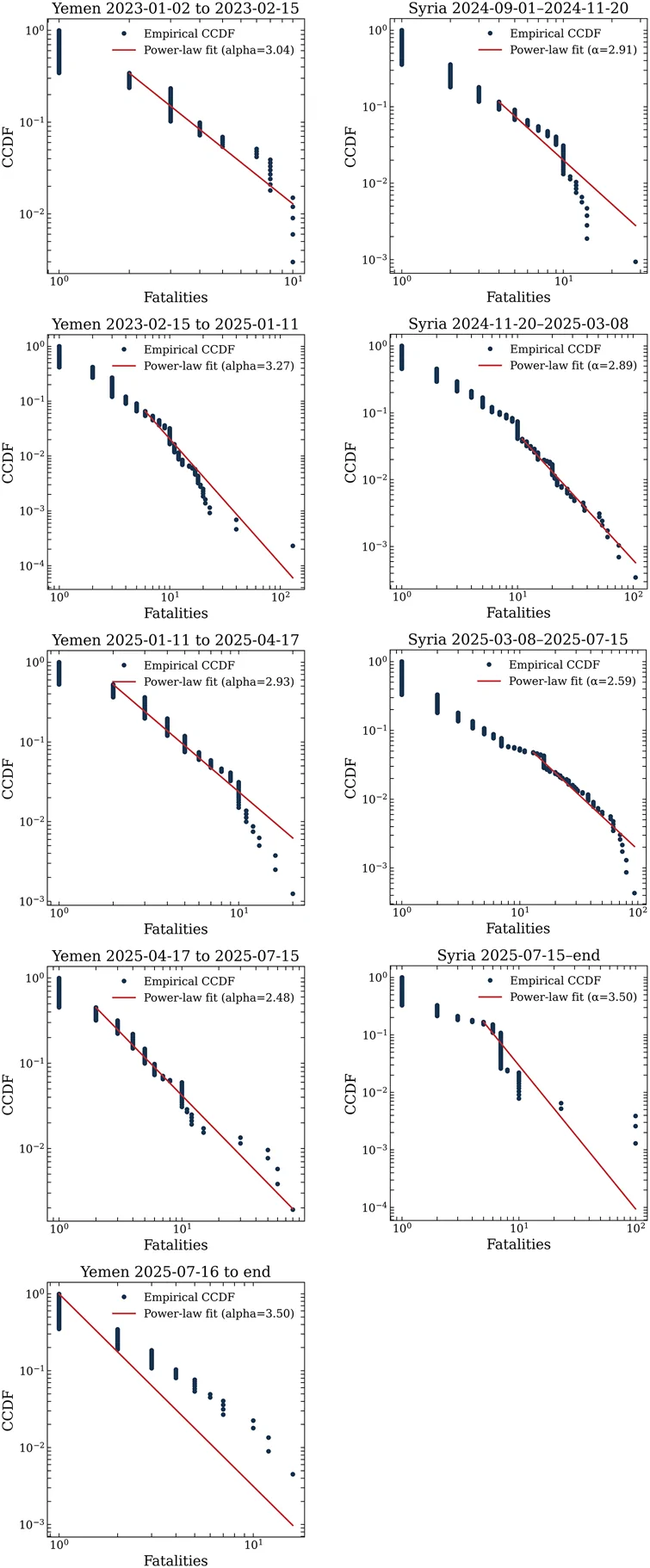
Empirical CCDFs and fitted power-law tails for the Yemen (left) and Syria (right) conflict periods analysed in tables 1 and 2. Blue markers denote the empirical CCDF, while red lines denote the fitted power-law tail above the estimated cut-off $x_{\min}$. The plots provide a visual goodness-of-fit diagnostic for the period-level power-law models and complement the KS statistics reported in the main text. Several periods exhibit reasonable agreement between the empirical and fitted tails, while others show visible departures that are consistent with the larger KS statistics and motivate the cautious interpretation adopted throughout the manuscript.

## Data Availability

Our paper uses existing data/models published elsewhere on publicly accessible websites, i.e. ACLED event-level conflict data cited in the main paper, the fusion–fission mathematical model cited in the main paper and already published, and the power-law code cited in the paper. All are publicly accessible.

## Appendix A. Fusion–fission generalizations

This brief discussion does not affect the main empirical results, but it provides additional theoretical context. Let $n_{s}(t)$ denote the number of fighter clusters of size s at time t. In the baseline fusion–fission model, fighter clusters evolve through fusion and fission events such that the total number of fighters is approximately conserved: new fighters can appear, and others can be removed as long as the churn is small compared with the overall number of fighters. Fusion events increase fighter-cluster size through the merging of smaller clusters, while fission events fragment clusters into smaller units. The mean-field evolution of $n_{s}(t)$ follows balance equations of the form

(A 1) $$\frac{\text{d}n_{s}}{\text{d}t}=F_{s}(\{n\})-D_{s}(\{n\}),$$

where $F_{s}$ and $D_{s}$ represent the rates of formation and destruction of fighter clusters of size s, respectively.

Under broad conditions, the stationary solution yields a power-law fighter-cluster-size distribution

(A 2) $$P(s)∼s^{-\alpha },$$

with tail exponent α determined by the relative dominance of fusion versus fission processes. More generally, fighters may be heterogeneous, belonging to distinct types indexed by $i=1,\ldots ,d_{S}$, representing differences in organization, ideology or allegiance. In this case, the fighter-cluster state is represented by a vector $s=(s_{1},\ldots ,s_{d_{S}})$, and cluster populations evolve according to coupled balance equations in a $d_{S}$-dimensional state space. Provided that post-fission fragments remain small relative to the total system size, the aggregate cluster-size distribution retains a power-law tail, with an effective exponent governed by the same fusion–fission balance as in the single-type case. The net result remains the prediction that α∼2.5+δ, where −1≲δ≲1 and the specific value depends on the relative robustness of larger fighter clusters within that particular conflict. Many extensions of the model therefore do not alter the primary empirical prediction of a power-law distribution with α in a range around 2.5.

## Appendix B. Complementary cumulative distribution function

To complement the KS diagnostics listed in tables 1 and 2, figure 3 provides visual goodness-of-fit assessments using CCDFs. For each period-level fit, the empirical CCDF is shown together with the fitted power-law tail above the estimated cut-off $x_{\min}$. These plots are intended as descriptive diagnostics rather than formal statistical tests and are included to provide additional transparency regarding the quality of the power-law fits.
